# Services for adults with ADHD: work in progress^†^: Commentary on . . . Specialist adult ADHD clinics in East Anglia

**DOI:** 10.1192/pb.bp.114.048850

**Published:** 2015-06

**Authors:** David Coghill

**Affiliations:** 1 University of Dundee, UK

## Abstract

Magon and colleagues highlight a number of relative strengths and weaknesses very reminiscent of those we have seen over the years in the development of similar services for children and adolescents. It is clear that we all have a lot of work to do to improve our approach to the transition from child to adult services. There was clear evidence that adult services can adapt to manage ADHD, but there is also a clear need for increased upskilling of clinicians in the practical management of medication and other treatments. I disagree with Magon and colleagues about the role of primary care and believe that treatment initiation and ongoing monitoring should, for the time being, remain in secondary care and that, because of the volume of work that will come our way, this will need to become a core rather than specialist task. As with other aspects of psychiatric care, there is a clear role for specialist nurses in delivering a significant proportion of the core care.

Magon and colleagues^1^ are to be congratulated for conducting what I believe is the first evaluation of the National Institute for Health and Care Excellence (NICE) recommendations on services for adults with attention-deficit hyperactivity disorder (ADHD). Their audit highlights several of the important, and sometimes controversial, issues that face commissioners, practitioners and patients.

This is a field of clinical work that is constantly evolving. Many of the changes that we are seeing mirror those that have taken place over the past two decades within child and adolescent mental health services (CAMHS). It will therefore be important that the lessons learnt there are not ignored. However, there are also many differences between the needs of people still experiencing ADHD and its related impairments as adults and those that they had while growing up and these too need to be respected. It is an important starting point to acknowledge that ADHD does not arise *de novo* in adulthood. There are of course some individuals with ADHD who were provided with ample scaffolding and support at home and school during childhood and adolescence and whose ADHD-related impairments may only have become clear when they left home and/or moved to less supported educational or work situations in early adulthood. But for most, their ADHD will have been causing significant problems throughout life and will have had an impact throughout their lives on their educational achievements, ability to form and sustain relationships, personality development and mental well-being.

## ADHD diagnosis rates in the UK

Unfortunately, in the UK at least, many of those with ADHD will not have received a diagnosis or any treatment during childhood or adolescence. Recent figures from Scotland suggest that despite increases in recognition over the past 20 years ADHD is still significantly under-diagnosed. Even in the regions with the highest rates of recognition only around one in five of those with ADHD are currently being diagnosed.^2^ There is also very significant variation between regions and in the most densely populated areas of Scotland the rates of recognition are even lower, with diagnosis and treatment rates running between 6 and 13% of the epidemiological prevalence.^2^ This same situation, both with respect to under-recognition and regional variability, is almost certainly replicated across the rest of the UK.

As a consequence, it seems very likely that those individuals who are being identified, diagnosed and treated are those with the most severe symptoms and impairments. They are also the group that is most likely to need continuing services and the greatest support during transition. It comes as no surprise to me to hear that transition was generally not well managed in either of the services covered in the audit and I recognise many of the comments in the discussion about the problems of transition only too well from my own clinical experience. From the CAMHS perspective it is often difficult to have the conversation about what the young person should expect from adult services when you are fully aware that in reality this depends a great deal on who picks up the case. It is much easier to hold on to cases well past the official transition date but this is neither good clinical practice – the needs of young adults are different and deserve to be treated by adult-oriented services – nor a good way to develop these much needed services.

## Guidelines for service transition

Acknowledging the challenges of developing these, essentially new, services, we have produced guidance for the establishment of transition services.^3^ These built on NICE recommendations^4^ and include suggestions that:

- a planned transfer to an appropriate adult service should be made if the young person continues to have significant symptoms of ADHD or other coexisting conditions that require treatment;
- transition should be planned in advance by both referring and receiving services;
- transition between teams should be a gradual process, e.g. lasting a minimum of 6 months;
- clear transition protocols should be developed jointly by commissioners, CAMHS/paediatric services, adult mental health services (AMHS) and primary care to facilitate transition and ensure that standards of care are maintained during the transition period;
- pre-transition: young people with ADHD should be reassessed at school-leaving age by the service managing their care; they should be informed of the outcome of this assessment and transitioned according to need;
- during transition, child and adult services should ideally have a joint transition appointment; during these meetings both services must ensure the needs of the young person will be appropriately met, which may involve further discussion and collaboration with educational and/or occupational agencies;
- post-transition: a comprehensive assessment should be carried out by the receiving service and this should include a reassessment for comorbid conditions;
- shared-care arrangements between primary and secondary care services for the prescription and monitoring of ADHD medications should be continued into adulthood.

Admittedly, these may seem to be relatively demanding recommendations, but the ongoing burden will be considerably lessened if they are underpinned by good-quality commissioning and planning. Getting the transition arrangements right should help to set the right tone for broader service planning. It should also strengthen the relationships between the child and adult services and facilitate joint learning, protocol development and planning.

## ADHD pharmacological treatment: competence issues

In contrast to the clear issues hindering transition, it was very encouraging to see that both audited services were generally doing very well with respect to the assessment of not only the core ADHD symptoms but also the physical needs and coexisting conditions as well as in the initiation of medication treatment. This clearly indicates that it is possible for adult services to develop the required skills and integrate them into day-to-day practice. Although I do not believe this was ever in doubt, it is not uncommon to hear colleagues say that they feel out of their depth when dealing with these patients. There are now increasing opportunities for training in the assessment and management of adults with ADHD. In particular, the UK Adult ADHD Network (UKAAN; www.ukaan.org) has developed, and can deliver, high-quality training within the UK and has produced a helpful handbook for clinicians.^5^ There are also recently updated guidelines from the British Association of Psychopharmacology.^6^

It has become clear in recent years that the pharmacological management of ADHD is a skilled task and that whereas the basic principles of treatment are relatively simple, it is not so easy to ensure that treatment is first optimised and then continues to work well over time.^7,8^

### Primary- *v.* secondary-care management

Various opinions have been proposed about why it is difficult to maintain treatment results over time, but it would seem that continuing to provide ongoing high-quality care may be one important part of the puzzle (e.g. see Langley *et al*^9^). This certainly seems to be the case in our own clinic and has led to us developing a nurse-led clinical pathway that focuses on providing high-volume, high-quality continuing care.^10^ This highlights the one area where I think I may disagree with Magon *et al*. I do not believe that primary care services in the UK have yet acquired the skills to manage ADHD independently of specialist services. I certainly fully support the use of shared-care arrangements whereby (post-stabilisation) prescribing and possibly some physical monitoring is managed in primary care. I strongly believe, however, that symptom monitoring, monitoring of impairment and functioning and monitoring of comorbid conditions should at present remain within secondary care. These are skilled tasks and take time to do properly, and even if general practitioners (GPs) acquire the skills they are unlikely to be able to spend enough time to ensure outcomes are optimised.

### Specialist ADHD management teams: (not) a perfect approach?

Although I do not currently support primary care-led services, it is still an open question as to which secondary care service model is best suited to the delivery of services for adults with ADHD in the National Health Service (NHS). Several different models have been put forward, ranging from all-age specialist teams, through specialist services limited to adults (such as those described in Magon *et al*’s audit), to services using a standardised protocol to deliver care via general AMHS. It is easy to see the allure of specialist teams, whether they be all-age or separate teams for children and adolescents and adults. There are, however, risks with this specialist team model. These include continued marginalisation of ADHD with specialist teams being seen as elitist and allowing those who are sceptical about ADHD to continue to ignore it as a valid clinical entity. Indeed, within such a model even those who may be somewhat interested but have not been placed on the team will often feel deskilled and left out.

Specialist teams often depend on a small group of individuals often centred around a charismatic and motivated leader. This makes them vulnerable when someone retires or leaves for another post. If successful, such teams will need to either become very big or only manage a small area. If they are large they can be very difficult to manage, if small one often gets postcode prescribing. On the other hand, expecting all adult psychiatrists to accept and manage ADHD alongside their other duties from the outset is almost certainly unrealistic and would be very likely to result in a huge variability in services. It would therefore seem parsimonious to accept that, as recommended by NICE, specialist services for adults with ADHD should be encouraged in the short to medium term, but that in the longer term it should be expected that managing ADHD will become a core skill for adult psychiatry, as it has over the past 20 years for child and adolescent psychiatry.

## A new way of delivering services

While the optimal configuration of services is still being debated, I strongly support the use of multidisciplinary skills in the delivery of care. It is certainly not the case that all clinical care needs to be delivered by a doctor. In Dundee we have successfully developed a pathway that is primarily delivered by trained nursing staff, most of whom are not prescribers. These skilled nurses run parallel clinics, often as many as five at a time, supported by a senior medical staff member (usually a consultant), who works as a ‘floating doctor’ across the clinics providing support as required and overseeing changes in medication. I am aware that several adult services have also been successful in adopting a similar nurse-led service model and believe this is the way forward for ADHD services across the age range. Clinical psychologists, occupational therapists, dieticians, a range of psychological therapists and voluntary sector staff can also play a very important role in providing holistic care and their involvement should be encouraged wherever possible. It is not essential, or often desirable, for everyone to be part of a physical team and there can be great benefits from adopting a ‘virtual team’ approach with a clear and shared clinical pathway.

Another important point raised by the audit is the current difficulty accessing non-pharmacological treatments. It is true that we do not yet have a solid evidence base for non-pharmacological approaches to the management of ADHD in adults, nevertheless I believe that NICE was correct to highlight the potential importance of broader approaches to treatment. As pointed out by Magon and colleagues, there are now a range of psychological therapies, most of which use a cognitive–behavioural therapy (CBT) approach. The problem seems to be getting those with the skills to deliver such programmes actually interested in doing so. Similar problems have arisen in effectively delivering behavioural parenting approaches. Here one solution has been to engage partners from outside the healthcare spectrum, often third-sector voluntary agencies, in delivery. It is not yet clear how this will work out for adult services. It may be the case that we have to wait until more clinical psychologists and/or appropriately trained nurse practitioners are ready to step up to the plate and start to provide a sound base of practice skills from which work can expand. Or it may be that, as suggested by Magon and colleagues, opportunities will arrive through the development of stronger and more active local ADHD support groups working in partnership with the voluntary sector.

The last point I would like to pick up on is that there were quite a few instances where the practice of the two audited services differed considerably. This is in line with the clear geographic differences in provision of services to children and young people. However, even though some degree of variability between services is of course inevitable, in ADHD very high levels seem to be the norm. Some of this variability will represent historical differences based on individual or service-level beliefs about the validity of ADHD and the use of medication to treat it and will have predated the provision of clear clinical guidelines. However, the introduction of guidelines does not seem to have led to increased uniformity in service delivery for child services^2,11^ and I suspect it will take a lot of hard work to ensure greater uniformity in the development of services for adults. Audits such as this are a good starting point and I again thank Magon and colleagues for getting the ball rolling.
